# Natural carbon fixation and advances in synthetic engineering for redesigning and creating new fixation pathways

**DOI:** 10.1016/j.jare.2022.07.011

**Published:** 2022-07-30

**Authors:** Sulamita Santos Correa, Junia Schultz, Kyle J. Lauersen, Alexandre Soares Rosado

**Affiliations:** a Laboratory of Molecular Microbial Ecology, Institute of Microbiology, Federal University of Rio de Janeiro, Rio de Janeiro 21941-902, Brazil; b Red Sea Research Center (RSRC), King Abdullah University of Science and Technology (KAUST), Thuwal 23955-6900, Saudi Arabia; c Computational Bioscience Research Center (CBRC), King Abdullah University of Science and Technology (KAUST), Thuwal 23955-6900, Saudi Arabia; d Bioengineering Program, Biological and Environmental Sciences and Engineering Division (BESE), King Abdullah University of Science and Technology (KAUST), Thuwal 23955-6900, Saudi Arabia; e Bioscience Program, Biological and Environmental Sciences and Engineering Division (BESE), King Abdullah University of Science and Technology (KAUST), Thuwal 23955-6900, Saudi Arabia

**Keywords:** Carbon fixation, Autotrophic, Thermophiles, Omics, Biochemistry, Synthetic pathway

## Abstract

**Background:**

Autotrophic carbon fixation is the primary route through which organic carbon enters the biosphere, and it is a key step in the biogeochemical carbon cycle. The Calvin–Benson–Bassham pathway, which is predominantly found in plants, algae, and some bacteria (mainly cyanobacteria), was previously considered to be the sole carbon-fixation pathway. However, the discovery of a new carbon-fixation pathway in sulfurous green bacteria almost two decades ago encouraged further research on previously overlooked ancient carbon-fixation pathways in taxonomically and phylogenetically distinct microorganisms.

**Aim of Review:**

In this review, we summarize the six known natural carbon-fixation pathways and outline the newly proposed additions to this list. We also discuss the recent achievements in synthetic carbon fixation and the importance of the metabolism of thermophilic microorganisms in this field.

**Key Scientific Concepts of Review:**

Currently, at least six carbon-fixation routes have been confirmed in Bacteria and Archaea. Other possible candidate routes have also been suggested on the basis of emerging “omics” data analyses, expanding our knowledge and stimulating discussions on the importance of these pathways in the way organisms acquire carbon. Notably, the currently known natural fixation routes cannot balance the excessive anthropogenic carbon emissions in a highly unbalanced global carbon cycle. Therefore, significant efforts have also been made to improve the existing carbon-fixation pathways and/or design new efficient *in vitro* and *in vivo* synthetic pathways.

## Highlights

• Carbon is often negatively associated with the anthropogenic emissions of greenhouse gases and, consequently, with environmental issues such as global warming. However, carbon is an element of paramount importance for life and ecological processes.
• The knowledge of carbon fixation cycles, in addition to the ecological and evolutionary studies on the emergence of the first beings that inhabited Earth and metabolized carbon, provides a detailed understanding of the enzymes and important intermediaries involved in these cycles.
• The central carbon metabolizing enzymes can be used for a variety of purposes; artificial CO_2_ fixation pathways can be designed and implemented in suitable host organisms; new solutions are possible for manipulating carbon fixation pathways and increasing their final yields.
• The multidisciplinary integration of omics, synthetic biology, molecular biology, and biochemistry can enable the scientific community to describe new carbon fixation pathways and predict important routes and enzymes involved in these processes. This integration may further provide guidance for overcoming the limitations of natural carbon fixation pathways and allow the development of biotechnological applications.
• Investigating thermophilic microorganisms is crucial for understanding many evolutionary events. These microorganisms are investigated extensively for developing biotechnological applications because of their metabolic diversity.
• In addition to the six well-known natural carbon fixation pathways, the development of omics and systems biology has spearheaded the discovery of new natural and synthetic pathways for carbon metabolism.

## Graphical abstract

Carbon circulation on Earth. Anthropogenic and natural carbon emissions and carbon sequestration routes.

**Figure:**
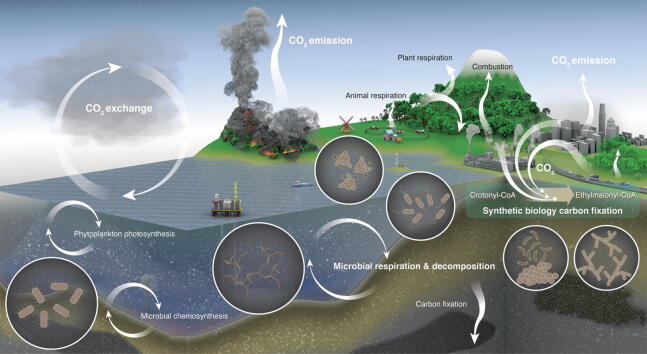

## Introduction

Carbon is the fourth-most abundant chemical element on Earth, after hydrogen, oxygen, and helium [1], [2], and it is considered as a base element for building organic compounds that are essential for life, such as proteins, carbohydrates, nucleic acids, and lipids. This element drives whole communities of living organisms and underpins the biogeochemical cycles on Earth [3].

**Table 1:** Comparative description of the natural and synthetic carbon fixation pathways: microorganism examples, energy sources, necessary inputs, products of the cycle, reducing agents, main enzymes, and sensitivity of the enzymes to oxygen.

***Pathway***	***Status***	***Organisms***	***Energy Source***	***Input***	***Output***	***Reductants***	***Key Enzyme***	***O_2_ Sensitive***	***References***
**Calvin-Benson**	*Natural*	Plants, Algae, Cyanobacteria, Aerobic Proteobacteria, Purple bacteria	Light	3 CO_2_, 9 ATP,6 NAD(P)H	Glyceraldehyde-3-phosphate	NAD(P)H	RuBisCO	No	[23]
**rTCA**	*Natural*	Green sulfur bacteria, Proteobacteria, *Aquificae, Nitrospirae*	Light and Sulfur	2 CO_2_, 2 ATP,4 NAD(P)H	Pyruvate	NAD(P)H and ferredoxin	2-Oxoglutarate synthase, Isocitrate dehydrogenase	Yes	[54]
**Wood–Ljungdahl**	*Natural*	Acetogenic, Methanogenic Archaea, Planctomycetes, Sulfate, *Archaeoglobales*	Hydrogen	2 CO_2_, 1 ATP, 4 NAD(P)H	Acetyl-CoA	Ferredoxin	NAD-independent formate dehydrogenase, Acetyl-CoA synthase-CO dehydrogenase	Yes	[9]
**3-HP**	*Natural*	*Chloroflexaceae*	Light	3 HCO_3_^−^, 5 ATP, 5 NAD(P)H	Pyruvate	NAD(P)H	Acetyl-CoA carboxylase, Propionyl-CoA carboxylase	No	[72], [187]
**HP/HB**	*Natural*	Aerobic *Sulfolobales*	Hydrogen and Sulfur	2 HCO_3_^−^, 4 ATP, 4 NAD(P)H	Acetyl-CoA	NAD(P)H	Acetyl-CoA-Propionyl-CoA carboxylase	No	[78]
**DC/HB**	*Natural*	Anaerobic *Thermoproteale, Desulfurococcales*	Hydrogen and Sulfur	1 CO_2_, 1 HCO_3_^−^, 3 ATP, 4 NAD(P)H	Acetyl-CoA	NAD(P)H and Ferredoxin	Pyruvate synthase, PEP carboxylase	Yes	[79]
**RHP**	Candidate *Natural*	*Methanospirillum hungatei*	Hydrogen	CO_2_, 3 ATP, 2 NAD(P)H	Gluconeogenesis and glycolysis	NAD(P)H	RuBisCO	No	[11]
**Natural Reductive Glycine**	Candidate *Natural*	*Candidatus phosphitivorax anaerolimiDesulfovibrio desulfuricans*	Phosphite	CO_2_, ATP, NAD(P)H	Formate/ Pyruvate	NAD(P)H and ferredoxin	CO_2_-reducing formate dehydrogenase (fdhAB)	–	[92]
**Reverse oTCA**	Candidate *Natural*	*Desulfurella acetivorans*	Hydrogen	CO_2_, ATP, NAD(P)H	Acetyl-CoA	Ferredoxin	Citrate synthase	–	[12]
**CETCH**	*Synthetic*	Theoretical	–	2 CO_2_, 2 ATP, 3 NAD(P)H	Glyoxylate	NAD(P)H	CoA- dependent carboxylase	No	[104]
**Reductive Glycine**	*Synthetic*	Demonstrated in *E. coli* as host	–	CO_2_, NADH	Pyruvate	Ferredoxin	Glycine cleavage system	No	[93]
**Synthetic malyl-CoA-glycerate**	*Synthetic*	Demonstrated in *E. coli* and *Synechococcus elongatus* PCC7942 host	–	CO_2_, 3 ATP, 3 NADH	Acetyl-CoA	NAD(P)H	PEP-carboxylase, RuBisCO	No	[119]
**SACA Pathway**	*Synthetic*	Demonstrated in *E. coli* as host	–	CO_2_	Acetyl-CoA	–	NAD-independent formate dehydrogenase	No	[6]
**Formolase pathway**	*Synthetic*	Theoretical	–	CO_2_, NADH, ATP	Dihydroxyacetone-phosphate	NADH	NAD-independent formate dehydrogenase	No	[5]

**Fig. 1:**
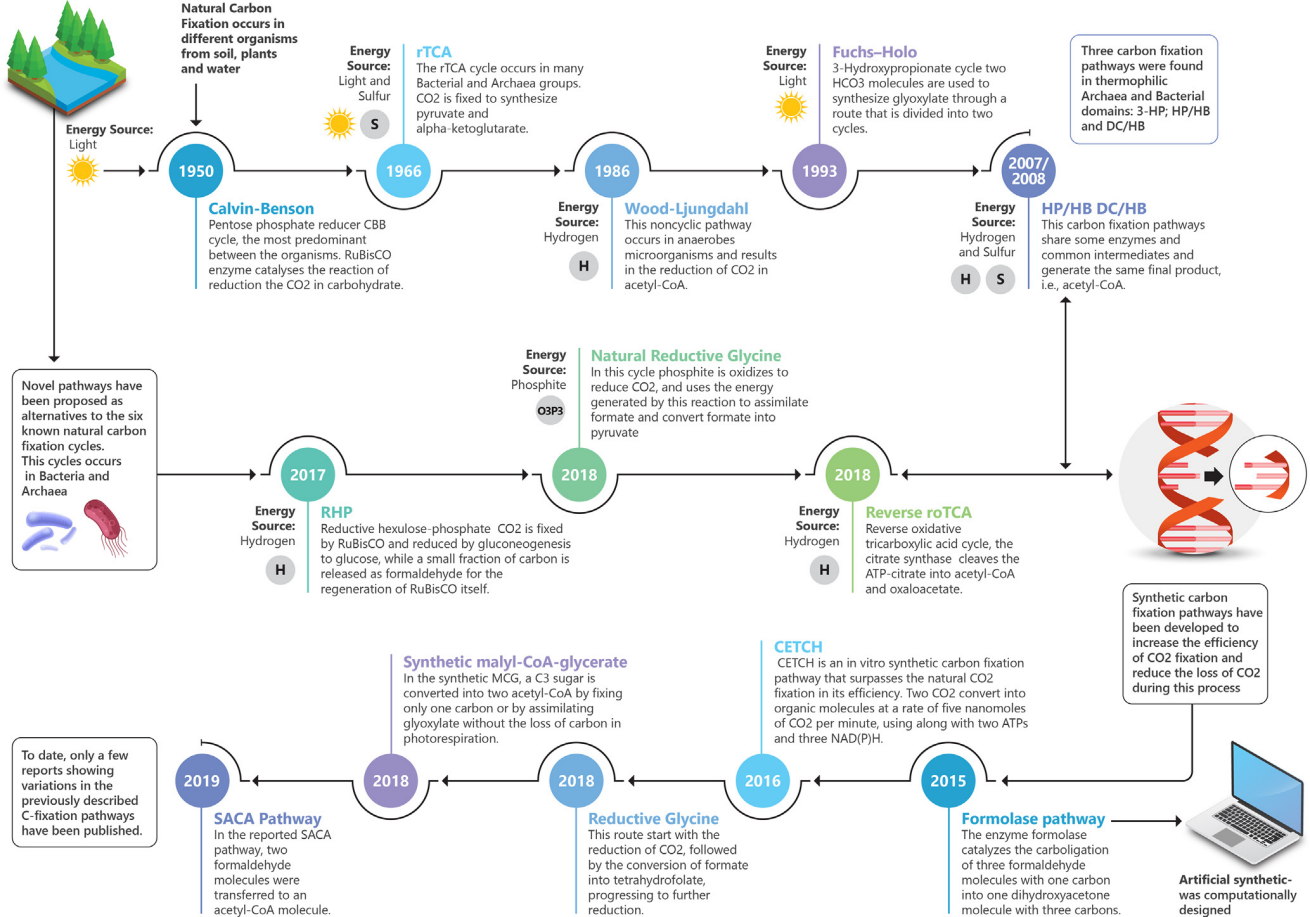
A timeline summary showing key milestones and findings related to carbon fixation pathways discussed in our work.

The entry of inorganic carbon into the biosphere is related to carbon fixation. Primary production of organic compounds is directly related to autotrophic carbon fixation, which converts inorganic carbon into biomass [4] (Fig. 1). At present, six natural autotrophic carbon-fixation pathways have been described and accepted, while other candidate routes have been recently proposed. With advances in synthetic biology, bioinformatics, and biochemical analyses, various synthetic carbon-fixation routes have also been developed (see Table 1), including the crotonyl-(CoA)/ethylmalonyl-CoA/hydroxybutyryl-CoA (CETCH; here, CoA represents coenzyme A) cycle, reductive glycine, synthetic malyl-CoA-glycerate pathway, synthetic acetyl-CoA (SACA) cycle, and formalase pathway [5], [6]. A timeline summary showing the key carbon fixation milestones discussed in our review is listed in Fig. 1.

The Calvin–Benson–Bassham (CBB) cycle is the most well-kwons mechanism of carbon assimilation and is found in all types of plants and algae and some prokaryotes [7]. However, new pathways of carbon fixation have also been elucidated in recent ecological, biochemical, and genomic studies [8]. In addition to the CBB cycle, autotrophic microorganisms can incorporate the carbon in biomass through five other natural carbon-fixation pathways: i) the reductive tricarboxylic acid (rTCA) cycle; ii) the reductive acetyl-CoA (also called the Wood–Ljungdahl [WL]) pathway; iii) the 3-hydroxypropionate [3-HP] bicycle; iv) the 3-hydroxypropionate/4-hydroxybutyrate (3-HP/4-HB) cycle; and v) the dicarboxylate/4-hydroxybutyrate (DC/4-HB) cycle [9], [10]. Three previously unknown natural carbon-fixation routes have also been described recently: i) the reductive hexulose-phosphate pathway (RHP), ii) the natural reductive glycine (nrGly) cycle, and iii) the reverse oxidative TCA cycle (roTCA) [11], [12].

Plants and photoautotrophic microorganisms are capable of fixing inorganic CO_2_ via the CBB cycle, which uses energy in the form of NAD(P)H and adenosine tri-phosphate (ATP) derived from photons. The global incident solar energy is 178,000 TW y^-1^, which is severalfold higher than that required by human society [13]. Oxygenic phototrophs, such as cyanobacteria, plants, and algae, have the ability to use this incident solar energy as a free source for photosynthesis-induced energy generation. Due to the release of oxygen in the process, these organisms are considered as the primary producers of the current biosphere [14]. Conversely, anoxygenic photosynthetic bacteria are also able to fix CO_2_ and grow autotrophically, typically using light as energy (ATP) and without producing oxygen in the process [15].

However, some autotrophic microorganisms are also capable of fixing inorganic carbon using other energy sources [16], [17]. Autotrophic organisms assimilate inorganic carbon into biomass using processes that are driven by light or chemical energy derived from organic molecule breakdown. Chemolithoautotrophic microbes use inorganic compounds (CO, CO_2_, H_2_, sulfate, phosphite, and Fe) as their energy sources, whereas chemoorganotrophic microorganisms rely on the energy produced from chemical transformations using organic compounds (sugars, organic acids, and amino acids) as carbon sources [17], [18].

Autotrophic organisms are crucial components of the global carbon cycle since they assimilate the organic carbon that is released by other organisms in the biosphere [8], [9], [10], [11], [12], [13], [14], [15], [16], [17]. The fossil fuel reserves that are essential in the current linear global economy represent carbon stored from the primary autotrophic production of biomass and sequestered in geological deposits. However, the unsustainable rates of global anthropogenic carbon release have now surpassed the ability of natural carbon-fixation pathways to recapture and fix this carbon in the biosphere [19]. Thus, an understanding of the metabolic conversion of inorganic carbon into organic carbon is critical for developing potential alternative solutions for capturing and using these waste carbon sources.

**Fig. 2:**
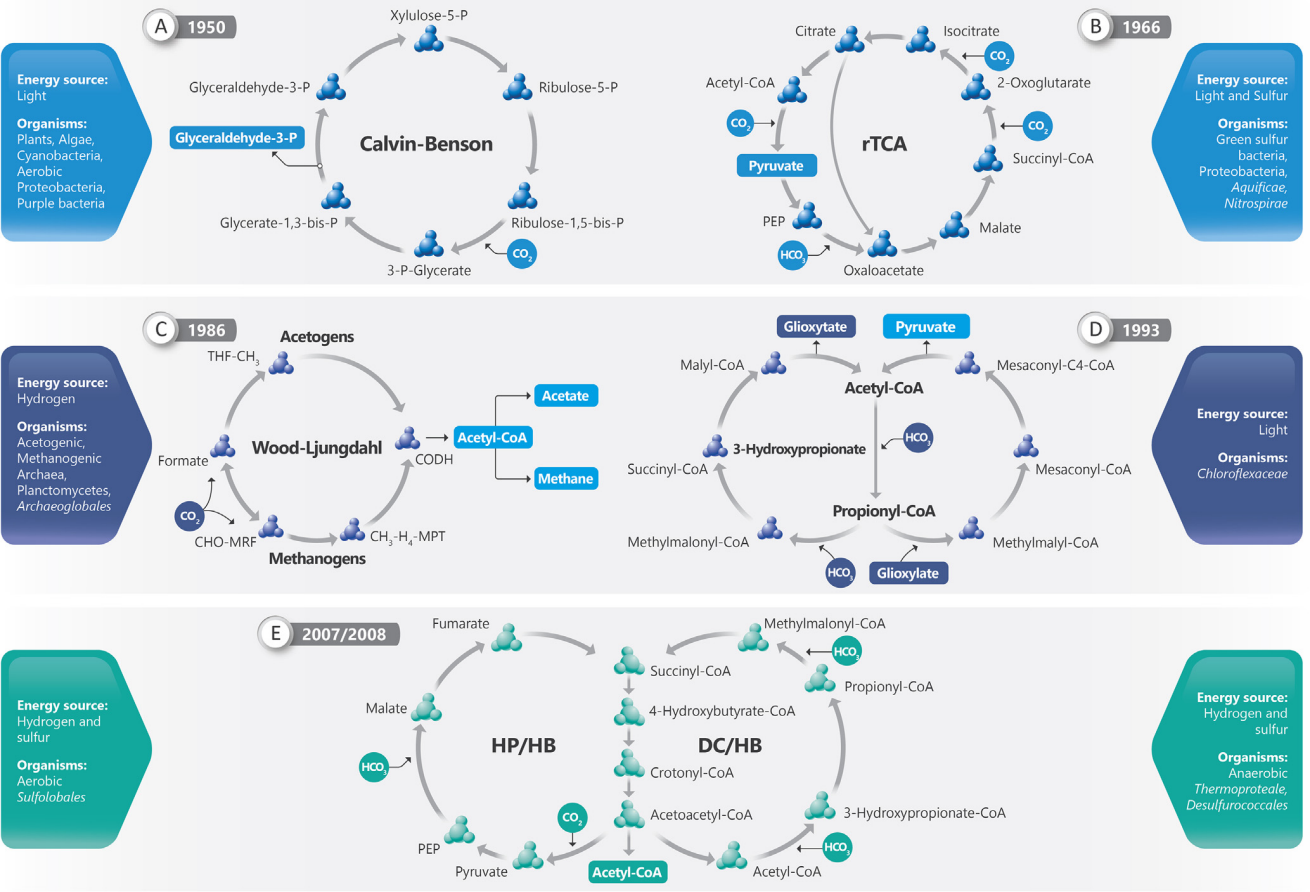
Natural carbon fixation. **(A)** CBB cycle; enzymes: ribulose-1,5-bisphosphate carboxylase/oxygenase, 3-phosphoglycerate kinase, glyceraldehyde-3-phosphate dehydrogenase, ribulose-phosphate epimerase. **(B)** rTCA cycle; enzymes: ATP-citrate lyase, malate dehydrogenase, succinyl-CoA synthetase, ferredoxin (Fd)-dependent-2-oxoglutarate synthase, isocitrate dehydrogenase, PEP carboxylase. **(C)** Wood–Ljungdahl cycle, upstairs acetogens Archaea and downstairs methanogens Archaea; enzymes: MPT-methylene tetrahydromethopterin, MFR-methanofuran, THF, tetrahydrofolate. **(D)** 3HP cycle; enzymes: acetyl-CoA carboxylase, propionyl-CoA carboxylase, methylmalonyl-CoA epimerase, succinyl-CoA:(*S*)-malate-CoA transferase, trifunctional (*S*)-malyl-CoA, -methylmalyl-CoA, mesaconyl-CoA transferase, mesaconyl-C4-CoA hydratase. **(E)** HP/HP and DC/HP cycle; enzymes: pyruvate synthase, PEP-carboxylase, malate dehydrogenase, fumarate hydratase/reductase, acetyl-CoA/propionyl-CoA carboxylase, 3-hydroxypropionate-CoA ligase/dehydratase, methylmalonyl-CoA mutase, succinyl-CoA reductase, 4-hydroxybutyrate-CoA ligase, crotonyl-CoA hydratase, acetoacetyl-CoA-ketothiolase.

The collective techniques that generate “omics” data on a massive scale are expected to facilitate the discovery of novel carbon-fixation pathways and efficient carbon-fixing enzymes [4], [5], [6], [7], [8], [9], [10], [11], [12], [13], [14], [15], [16], [17], [18], [19], [20]. Future trends are focused on manipulating the known autotrophic carbon-fixation pathways to increase their efficiency and to understand their components, as well as the development of new and more efficient natural and synthetic fixation cycles [21], [22]. Moreover, thermophilic microorganisms are also valuable sources of metabolic diversity and are currently being investigated for alternative carbon-fixation pathways, because a chemolithoautotrophic thermophile is probably the most common precursor to life and provides the best model for investigating primordial metabolism [7]. This review summarizes and discusses the six known autotrophic pathways of carbon fixation, the newly identified and proposed natural pathways, as well as recent advances in designing new pathways for biological and synthetic carbon fixation (Fig. 2). We discuss the continued progress of “omics” tools and their role in unveiling new routes of carbon fixation and the efforts devoted to identifying the enzymes that participate in carboxylation reactions. Furthermore, efforts to develop engineered enzymes with activities that surpass those found in natural carbon-fixation cycles are also discussed. Subsequently, we focus on carbon fixation performed by thermophilic Bacteria and Archaea to understand the carbon-fixation pathways and obtain insights for future attempts at synthetic engineering. Finally, we highlight the knowledge gaps in the field as well as the challenges and new directions for future studies on carbon fixation.

## Autotrophic carbon-fixation pathways: The six known cycles

### Calvin**–**Benson–Bassham cycle

The photosynthetic and pentose phosphate reducing pathway (CBB pathway) discovered by Melvin Calvin, Andrew Benson, and James Bassham in the 1940 s and 1950 s [23] was the first reported carbon-fixation pathway. The CBB cycle is present in algae, plants, cyanobacteria, phyla Proteobacteria and Firmicutes, and photoautotrophic and chemoautotrophic bacteria [24], [25]. In microorganisms, this cycle is the most important carbon dioxide fixation pathway [26]. The key enzymes in this pathway include ribulose-1,5-bisphosphate carboxylase/oxygenase (RuBisCO) and phosphoribulokinase (PRK) [27] (Fig. 2A). Another important enzyme in the CBB cycle is glyceraldehyde-3-phosphate dehydrogenase (GAPDH). GAPDH and PRK participate in the regeneration of RuBisCO and form an inactive complex with the regulatory protein CP12 when in a dark environment [28]. CP12 is formed by 80 amino acids and, after binding to GAPDH and PRK, becomes an important light/dark regulator [29]. In dark, the CP12 protein complex is deactivated, while under light conditions, this complex is dissociated, allowing the reactivation of GAPDH and PRK enzymes [30]. Most oxygenic photosynthetic organisms that use the CBB cycle to fix CO_2_ contain CP12, whereas nonoxygenic phototrophic bacteria do not have this protein [28], [29], [30]. In the CBB cycle, three molecules of CO_2_ are fixed by RuBisCO to form one molecule of glyceraldehyde 3-phosphate, in a pathway composed of a total of 29 reactions from 13 enzyme reactions [31]. RuBisCO drives the production of carbohydrates by catalyzing the removal of CO_2_ from the atmosphere and its conversion into carbohydrates for plants and other organisms [32].

Recently, a novel variant of the CBB cycle was described using computation of elementary flux modes or extreme paths to find all pathways leading to the formation of glyceraldehyde 3-phosphate from CO_2_ [31]. Named as the S7P-removing transaldolase variant, it involves transaldolase, and it bypasses the intermediate enzyme fructose 1,6-bisphosphate [31]. The author suggested that transaldolase can be an alternative for enhancing photosynthetic carbon metabolism. Previously, this theoretical path was proposed as the S7P-forming transaldolase variant because it requires an S7P-forming step [33], different from the variant described by Ohta [31]. The S7P-forming transaldolase variant works twice per three CO_2_ molecules fixed, in which transaldolase transfers three carbons from fructose 6-phosphate to erythrose 4-phosphate to make S7P and glyceraldehyde 3-phosphate [33], [34].

RuBisCO catalyzes both the carboxylation and oxygenation of its substrate [35]. CO_2_ and O_2_ compete for the same active site on RuBisCO when reacting with the same substrate. The carboxylation results in the formation of two molecules of a three-carbon-containing organic acid, 3-phosphoglycerate (3-PGA), whereas RuBisCO oxygenation produces one molecule of 3-PGA and another of 2-phosphoglycolate (2-PG) using a process called photorespiration [36]. The 3-PGA molecule resulting from RuBisCO carboxylation generates carbohydrates, whereas 2-PG is not metabolized in the CBB cycle and is instead used in respiration, wherein it consumes O_2_ and releases already fixed CO_2_ [37].

To increase the concentration of CO_2_ near RuBisCO and decrease the photorespiration rate, cyanobacteria and some chemoautotrophs contain an intracellular microcompartment–carboxysome–that functioning as an organelle [38]. The carboxysomes encapsulate RuBisCO and the carbonic anhydrases, and operate as semipermeable barriers that allow the passage of HCO_3_ and RuBisCO and exclude O_2_ that competes for the substrate. In the carboxysome, the carbonic anhydrases catalyze the conversion of HCO_3_ into CO_2_, which is fixed by RuBisCO to form 3-PGA [39]. The efficient CO_2_ fixation mechanism in the carboxysomes recently inspired their implementation in plants to increase crop productivity [40]. In some eukaryotic algae, carbon fixation is mediated by several mechanisms to enrich the CO_2_ concentration locally as the pyrenoid, which appears as a dynamic compartment of membranes and crystalline starch under a microscope [41]. A recent study provided the first glimpse of the cycle of a carboxysome inside living cells through a fluorescence imaging platform that allows the simultaneous measurement of the numbers, positions, and activities of carboxysomes in cyanobacteria [42].

## Importance of RuBisCO

Four forms of RuBisCO, which has a structure formed by eight large subunits and eight small subunits, are known [43]. The I and II forms participate in the autotrophic assimilation of CO_2_. RuBisCO form I is more widely distributed, and is found in eukaryotes and some prokaryotes. Additionally, form I RuBisCO is roughly 2.4 million years old and emerged from the great oxygenation event that occurred owing to the transforming action of cyanobacteria, which began to introduce oxygen into the atmosphere driven by the abundant photosynthetic energy provided by the sunlight. Meanwhile, RuBisCO form II occurs in prokaryotes (mostly in Proteobacteria species) and microeukaryotes such as dinoflagellates (i.e., *Symbiodinium*) [44]; these are the only groups capable of processing RuBisCO form II, which is encoded by the nuclear DNA [45], [46].

Banda et al. [47] discovered an uncharacterized clade sister of RuBisCO form I, i.e., RuBisCO I’. Their study involved the metagenomic analysis of samples from environmental communities with a large amount of nonculturable bacteria. They reported that RuBisCO form I’ may have evolved under anaerobic conditions before the evolution of cyanobacteria. Unlike RuBisCO form I, RuBisCO form I’ consists of only eight large subunits without the small subunits. Twenty-four *rbc*L genes with gene products that share a high sequence homology (52 %–61 %) with the known RuBisCO form I were reported. However, a more detailed analysis of the genomes assembled from the metagenome indicated the absence of the *rbc*S genes [47]. These genes are always found in Bacteria and Archaea that use the CBB cycle to fix carbon [48]. On the basis of these results, the group suggested that RuBisCO I’ probably represents a distinct form of RuBisCO that presumably diverged from form I before the origin of life.

According to previously reported studies, form III of RuBisCO does not participate in autotrophic CO_2_ fixation; instead, it uses ribonucleotides via the pentose-bisphosphate pathway [49]. However, using “omics” and biochemical approaches, Frolov et al. [25] identified an operative form III RuBisCO in a transaldolase variant of the CBB cycle in *Thermithiobacillus tepidarius* and *Thiobacillus thioparus*. Form III was found exclusively in Archaea [50], and since then, it has been hypothesized to exist in bacterial candidates [51].

RuBisCO form IV is known to not perform carboxylase and oxygenase activities and is distributed in seven phylogenetically distinct subgroups, namely, IV-Photo (phototrophic bacteria), IV-Nonphoto (nonphototrophic bacteria), IV-AMC (microbial consortia found in acid mines lakes), IV-GOS (ocean sampling sequences), IV-DeepYkr (deep branch close to the YkrW clade), IV-Aful (*Archaeoglobus fulgidus*), IV-YkrW (*Bacillus*) [52], and a recently discovered form that acts as an oxygenase that converts 3-keto-d-ribitol-1,5-bisphosphate into two hydroxy acid phosphorylates: 3-d-phosphoglycerate and phosphoglycolate [53].

## Reductive tricarboxylic acid cycle (rTCA)

The second carbon-fixation pathway was described in 1966 by Evans and collaborators [54]. This route was revealed in *Chlorobium limicola*, a green sulfur bacterium. Experiments with fermentative bacteria revealed a reaction that challenged the concepts of carbon fixation. Ferredoxin was found to serve directly as an electron donor for carbon fixation for the synthesis of pyruvate and alpha-ketoglutarate from CO_2_ and reduced ferredoxin and acetyl-CoA or succinyl-CoA, respectively, through an irreversible pathway [55].

This route was named rTCA or the Arnon–Buchanan cycle and is believed to be the most plausible candidate for the first autotrophic metabolism [56]. In the rTCA cycle, two cofactors mediate the carbon fixation: thiamine pyrophosphate, which converts acetyl-CoA to pyruvate, and succinyl-CoA to produce ketoglutarate [57]. This cycle behaves in a direction opposite to that of the oxidative citric acid cycle (oTCA; one of the stages of cellular respiration in heterotrophs, which is performed in the presence of O_2_ and releases fixed carbon) and forms acetyl-CoA from two CO_2_ molecules.

Some key enzymes of the Krebs cycle are considered to react irreversibly and are replaced in the rTAC cycle to reverse the cycle; for example, succinate dehydrogenase is substituted by fumarate reductase; NAD 2-oxoglutarate dehydrogenase is replaced by ferredoxin 2-oxoglutarate synthase; and citrate synthase is replaced by ATP-citrate lyase [54], [58], [59]. The main product of carbon fixation in the rTCA cycle is acetyl-CoA, which is later converted into other central intermediates of the carbon metabolism, including pyruvate/phosphoenolpyruvate (PEP), oxaloacetate, and 2-oxoglutarate [56]. Acetyl-CoA is carboxylated into pyruvate by ferredoxin-dependent pyruvate synthase, and pyruvate can also be converted into PEP; oxaloacetate is synthesized in the reactions of pyruvate or PEP carboxylase [54] (Fig. 2B).

The rTCA cycle occurs in many Bacteria and Archaea groups and includes enzymes that are sensitive to oxygen. For this reason, this cycle is observed in anaerobes or facultative anaerobes that can tolerate O_2_ concentrations lower than that found in the air [60]. Although discovered in photosynthetic green sulfur bacteria, the rTCA cycle has been shown to be present in plenty of chemoautotrophic microbes [55]. More recently, it was assumed that the complete TCA cycle can occur in *Pandoravirus massiliensis*, which showed genes with low similarity to all enzymes of the cellular TCA cycle and, most importantly, a functional isocitrate dehydrogenase, a key enzyme of this cycle [61]. The gene ORF132 that encodes isocitrate dehydrogenase was cloned and expressed in *Escherichia coli,* and was shown to encode an isocitrate dehydrogenase. The authors suggested the possible existence of an autonomous TCA pathway or another unknown metabolic pathway that may be involved in redirecting amoeba carbon metabolism where this virus was replicated [61].

## Reductive acetyl-CoA pathway

In 1986, Ljungdahl and collaborators [9] described the third carbon-fixation route, i.e., the reductive acetyl-CoA or WL pathway. This noncyclic pathway occurs in anaerobic microorganisms and results in the reduction of CO_2_ in acetyl-CoA. According to the reported studies, these microbes may have been the first autotrophs to use inorganic compounds such as CO and H_2_ as energy sources and CO_2_ as an electron acceptor one billion years before the appearance of O_2_ [62].

In this cycle, two CO_2_ molecules are converted into acetyl-CoA through two different branches that operate in parallel: the eastern or methyl branch and the western or carbonyl branch [63]. In the eastern branch, one CO_2_ molecule undergoes six-electron reduction to form a methyl group, whereas the western branch involves the reduction of the other CO_2_ molecule to CO, which is condensed with the methyl group and CoA to form acetyl-CoA [64]. Acetyl-CoA is then incorporated into cellular carbon or converted to acetylphosphate, from which the phosphoryl group is transferred to adenosine diphosphate (ADP) to generate ATP and acetate, which are the main growth products of acetogenic bacteria [9], [65], [66].

The eastern branch begins with the reduction of CO_2_ in formate catalyzed by the formate dehydrogenase enzyme, which undergoes ATP-dependent condensation with tetrahydrofolate (H_4_F) to form 10-formyl-H_4_folate, which is converted to 5,10-methylenyl-H_4_folate. The next step is the reduction of 5,10-methylene-H_4_folate to 5-CH_3_-H_4_folate [63]. At the end of the branch, the methyltetrahydrofolate (corrinoid/iron–sulfur protein) methyltransferase enzyme catalyzes the relocation of the methyl group on N5 of (6S)–CH_3_-H_4_folate to the cobalt center of the corrinoid iron-sulfur protein (CfeSP). In contrast, the western branch involves one enzyme, i.e., the CO-dehydrogenase/acetyl-CoA synthase, which catalyzes the conversion of CO_2_ into CO [67].

When organisms are cultivated with CO, CO dehydrogenase generates CO_2,_ which is converted into formate in the eastern branch, and CO is incorporated directly into the carbonyl group of acetyl-CoA. In the next step, the acetyl-CoA synthase catalyzes the condensation of CO, CoA, and the methyl group of a methylated CfeSP to generate acetyl-CoA; at this point, the western branch meets the eastern branch.

In the Archaea *Methanosarcina acetivorans*, the conversion of methanogen to acetogen has been shown to completely dispense with the conserved methanogenesis energy [68]. *Methanosarcina acetivorans* uses the reducing acetyl-CoA to conserve energy in the form of acetate [69]. The genes encoding Mtr, i.e., disruption from the methyl branch toward methane, were eliminated in *M. acetivorans,* and all carbon from the methanogenesis substrate was diverted to flow through acetyl-CoA [68]. Thus, *M. acetivorans* could be converted into an acetogenic organism, which suggests that methanogenesis may have evolved from the acetyl-CoA pathway [68].

The reductive acetyl-CoA route is found in prokaryotes that live at thermodynamic limits such as the acetogenic and methanogenic Archaea [23], which use this route not only for the reduction of CO_2_ in acetyl-CoA but also for the conservation of energy by generating an electrochemical gradient [70]. In the energy-conservation process occurring during autotrophic growth or in the fermentation process, the acetogens and methanogens generate acetate and methane, respectively [71] (Fig. 2C).

## 3-Hydroxypropionate bicycle

The 3-hydroxypropionate bicycle (or the Fuchs–Holo route) was found in *Chloroflexus aurantiacus*, which is a green sulfur thermophilic bacterium, by Helge Holo and Georg Fuchs in 1989 [58], [72]. In this pathway, two HCO_3_ molecules are used to synthesize glyoxylate through a route that is divided into two cycles. In the first cycle, glyoxylate is formed from the carboxylation of acetyl-CoA to produce malonyl-CoA, which is reduced to proprionyl-CoA via hydroxypropionate, after which the carboxylation of proprionyl-CoA produces succinyl-CoA. Subsequently, succinyl-CoA is converted to (S)-malyl-CoA, regenerating acetyl-CoA (starting molecule) and releasing glyoxylate as the first product of the carbon fixation. The second cycle begins with the assimilation of glyoxylate and its conversion to methylmalyl-CoA, which in turn is converted to form citramailyl-CoA by mesaconyl-CoA. Finally, the product citramailyl-CoA is converted into pyruvate and acetyl-CoA, thereby regenerating the precursors of the cycle [73] (Fig. 2D).

This pathway involves 19 steps and 13 enzymes; none of the steps are sensitive to oxygen and all the enzymes work under aerobic conditions [74]. This fourth carbon-fixation pathway appears to be exclusively restricted to a single clade of photosynthetic *Chloroflexus* [75]. This green sulfur bacterium uses the 3-hydroxypropionate bicycle in combination with the glyoxylate cycle to channel organic substrates such as glycolate, acetate, propionate, 3-hydroxypropionate, lactate, butyrate, or succinate for its central carbon metabolism under both autotrophic and heterotrophic growth conditions [76].

## Hydroxypropionate/4-hydroxybutyrate (HP/HB) cycle and dicarboxylate/4-hydroxybutyrate (DC/HB) cycle

The fifth and sixth carbon-fixation pathways share some enzymes and common intermediates and generate the same final product, i.e., acetyl-CoA [77] (Fig. 2E). The fifth carbon-fixation pathway—the 3-hydroxypropionate/4-hydroxybutyrate cycle (HP/HB) was described in *Metallosphaera sedula,* which is a thermophilic Archaea belonging to the Sulfolobales order [78]. The sixth pathway (dicarboxylate/4-hydroxybutyrate [DC/HB]) was found in *Ignicoccus hospitalis*, which is an Archaea belonging to the order Desulfurococcales [79]. Existing variants of both cycles, the orders Sulfolobales and Desulfurococcales, did not evolve from a common ancestor, and the representatives of these two orders have phylogenetically unrelated enzymes [80].

In these two cycles, two CO_2_ molecules are added to acetyl-CoA to produce succinyl-CoA and rearrange into acetoacetyl-CoA and cleave into two acetyl-CoA molecules [81]. In the HP/HB cycle, an acetyl-CoA and the propionyl-CoA carboxylase, fixes two HCO_3_ molecules, and in the DC/HB cycle, the pyruvate synthase and PEP carboxylase catalyze this reaction. These pathways vary mainly in relation to oxygen tolerance and the use of reduction cofactors: NAD(P)H is used in the HP/HB cycle, and ferredoxin/NAD(P)H is employed in the DC/HB cycle [23], [82]. The enzymes in the HP/HB cycle are oxygen-tolerant, whereas the 4-hydroxybutyryl-CoA dehydratase enzyme in the DC/HB cycle is inactivated in the presence of oxygen. Although the two cycles produce acetyl-CoA, their main difference lies in their link to the carbon metabolism. In the DC/HB pathway, pyruvate is synthesized from acetyl-CoA by using a pyruvate synthase, and in the HP/HB pathway, another half turn of the cycle is needed to produce succinyl-CoA, which is oxidized via succinate to produce pyruvate [83].

This cycle is also found in *Nitrosopumilus maritimus*, belonging to the order Sulfolobales. This species heterologously produces the protein Nmar_1028 that catalyzes the conversion of succinyl-CoA into two acetyl-CoA molecules. Nmar_1028 is homologous to the dehydrogenase domain of crotonyl-CoA hydratase/(S)-3-hydroxybutyryl-CoA dehydrogenase and seems to be the only (S)-3-hydroxybutyryl-CoA dehydrogenase in *N. maritimus* and is thus essential for the functioning of the 3-hydroxypropionate/4-hydroxybutyrate cycle [84]. In *Metallosphaera sedula*, Msed_2001 catalyzes the same reaction as Nmar_1028, and these two enzymes are homologous and have evolved independently from their respective bacterial homologs [15]. The existence of the HP/HB cycle in two distantly related archaeal groups with different physiologies is not evident in the course of evolution of autotrophic CO_2_ fixation, because this process would need to be adapted to the corresponding ecological niche [15].

## New candidate pathways for natural carbon fixation

Recent studies have revealed that carbon fixation is not limited to these six cycles (Tab. 1). Genome analyses have shown that several autotrophs do not present genes from any of the already described autotrophic CO_2_-fixation pathways [23]. Therefore, investigations performed in this direction are of utmost importance for establishing novel ways of fixing inorganic carbon and exploring alternative routes for metabolic energy supply [85].

## RHP pathway

To date, three novel pathways have been proposed as alternatives to the six known natural carbon-fixation routes. Kono et al. [11] fully described the RHP pathway in the methanogenic Archaea, *Methanospirillum hungatei*. The carbon metabolism has been shown to involve the enzymes RuBisCO and phosphoribulokinase (PRK), which are the same enzymes found in the CBB cycle. RuBisCO of *M. hungatei* seems to form a new clade with RuBisCO of another methanogenic Archaea that also has PRK; this clade is different from RuBisCO form III. The coexistence of the RuBisCO and PRK in this Archaea led the authors to propose that *M. hungatei* fixed carbon. However, the enzymes transketolase, ribulose-5-phosphate and sedoheptulose-1,7-bisphosphatase, which participate in the CBB cycle, are absent from the genome. Archaea are known to lack genes for a transketolase, which is essential in the CBB cycle [86].

The proposed RHP pathway is noncyclical and shares the majority of the reactions of the CBB cycle, except for those in the regeneration stage. In the RHP cycle, CO_2_ is fixed by RuBisCO and reduced by gluconeogenesis to glucose, while a small fraction of carbon is released as formaldehyde for the regeneration of RuBisCO itself [11]. Because the RHP cycle differs from the CBB cycle in some steps, the reported results provide insights into the evolutionary and functional associations between carbon metabolism and the pathways involving the RuBisCO enzyme present in photosynthetic organisms and methanogenic Archaea. The authors speculate that the CBB cycle and RHP originated from a primitive carbon metabolic pathway that used RuBisCO, but with the replacement of a few steps and without carbon release. Although the energy requirements of the RHP pathway are significantly lower than those of the CBB cycle in plants and cyanobacteria, it does not proliferate as in the case of the CBB.

## Natural reductive glycine (nrGly) pathway

Next-generation sequencing can provide a holistic approach to understand metabolisms through multiple “omics” datasets [87], [88]. The data generated by genomics, proteomics, transcriptomics, and metabolomics have significantly increased the understanding of cell physiology and provided answers to important questions about the metabolisms of microorganisms [88], [89]. Studies on the (meta)genome and (meta)transcriptome have also made it possible to identify the key genes involved in carbon fixation, allowing the prediction of potential carbon-fixation routes in (unculturable) environmental microbes as well as the discovery and design of new pathways for carbon fixation [90], [91].

Figueroa et al. [92] proposed another candidate carbon-fixation route, nrGly, using 16S rRNA region sequencing. The group proposed that inorganic carbon is assimilated into the glycine-reducing pathway found in the uncultivated bacterium *Candidatus Phosphitivorax anaerolimi* strain Phox-21, belonging to the class Deltaproteobacteria. This pathway was previously described as a synthetic route for carbon fixation [93]. In this proposed route, Phox-21 oxidizes phosphite to reduce CO_2_, and uses the generated energy to assimilate formate through the glycine-reducing pathway. Due to the lack of alternative electron acceptors, Phox-21 has been suggested to couple phosphite oxidation to CO_2_ reduction; in addition to the presence of CO_2_ reductase and formate dehydrogenase (FdhAB) in its genome, this assumption could explain the mechanism by which this metabolism occurs [92]. The bacteria reduce CO_2_ to formate by using FdhAB and convert formate into pyruvate via the glycine-reducing pathway. All the necessary precursors (acetyl-CoA, oxaloacetate, 2-oxoglutarate, and succinyl-CoA) can subsequently be generated from the pyruvate route in the partial rTCA cycle. The results of Phox-21 genomic analysis suggest that it may be possible for microorganisms to exploit the energy derived from phosphite oxidation, enabling autotrophic growth through the glycine-reducing pathway with CO_2_ as the only electron acceptor [92].

Because of its very low redox potential, phosphite might be the only biological electron donor that can drive the fixation of CO_2_ in chemotrophs through the reductive glycine pathway (an ATP-consuming network) in the absence of an additional energy source or electron acceptor. Furthermore, according to other reported studies, phosphite oxidation may be coupled with CO_2_ reduction through the WL pathway [94]. Although genomic analysis has indicated a new carbon-fixation pathway, experimental evidence is required to verify the validity of this hypothesis.

Sánchez-Andrea et al. [95] described the reductive glycine pathway in *Desulfovibrio desulfuricans*. In addition to performing genomic, transcriptomic, proteomic, and metabolomic analyses, they also performed *in vivo* evaluation of autotrophic growth of bacteria. Unlike *C. Phosphitivorax anaerolimi*, the results of the study on *D. desulfuricans* demonstrated the enrichment of the culture medium with an organic carbon compound (cysteine and ruminal fluid) via autotrophic growth. The studies performed with labeled CO_2_ and functional “omics” experiments proved the fixation of carbon through the reductive glycine pathway. The results of this study demonstrate that the reductive glycine pathway is the only carbon-fixation pathway that allows autotrophic growth of *D. desulfuricans*. In the reductive glycine pathway, firstly, CO_2_ is first reduced to formate, which is further reduced and condensed with a second CO_2_ molecule to generate glycine. The resulting product is then reduced by the glycine reductase to acetyl-P and then to acetyl-CoA, which is further condensed with another CO_2_ molecule to form pyruvate [95]. Thus far, it seems that this is the first reported study to prove the operation of the entire reductive glycine pathway in a microorganism.

Recently, Hao et al. [96] identified two *C. Phosphitivorax* strains (F81 and R76) as butyrate oxidizers. Transcriptome analyses were performed, and the entire *ptx*-*ptd* gene cluster for phosphite oxidation was reconstructed in strain F81; interestingly, this cluster was not found in strain R76. Genes involved in the glycine-reduction pathway were found in both the strains, whereas the essential genes for other autotrophic CO_2_ pathways were absent. However, according to the authors, although genes for the glycine-reduction pathway were observed, they were not expressed. This observation indicates that these microorganisms, unlike the Phox-21 strain that was externally enriched with CO_2_ and phosphate, live as heterotrophic syntrophs besides autotrophs. Other studies demonstrated that the glycine-cleavage system can support glycine and serine biosynthesis from formate in an engineered *E. coli* strain at increased CO_2_ concentration [97], [98]. Nonetheless, it is necessary to verify the growth of the bacterium on formate (and also CO_2_), which remains an unresolved challenge [99].

## Reverse oxidative tricarboxylic acid cycle (roTCA)

Although metagenomic analyses have gained considerable acceptance in the studies of organisms and microbial communities, researchers have reported the need for joint efforts involving both “omics” and biochemistry [12]. In studying *Desulfurella acetivorans,* which is a sulfur-reducing and thermophilic Deltaproteobacteria (optimal growth at 52 °C to 57 °C), the authors elucidated a new version of the rTCA cycle, which was named the reverse oTCA cycle (roTCA) (this report also mentions the oTCA cycle that provides energy for aerobic organisms and has citrate synthase as the key enzyme). The genome analysis of *D. acetivorans* revealed the absence of key genes and enzymes involved in the known pathways of carbon fixation normally found in organisms exhibiting autotrophic growth. On the basis of this information, the authors investigated *D. acetivorans* in detail using classical biochemical techniques. *D. acetivorans* can grow heterotrophically using acetate as an electron donor and carbon source [100] or autotrophically with H_2_ [101]. As previously described, the key enzyme in the rTCA cycle is the ATP-dependent citrate lyase enzyme; however, *D. acetivorans* do not have the genes that encode that enzyme.

In the roTCA pathway, citrate synthase is the key enzyme that cleaves ATP-citrate into acetyl-CoA and oxaloacetate. Using ultra-performance liquid chromatography, the cleavage of citrate into acetyl-CoA and oxaloacetate was observed in bacterial cell extracts, with the latter being further reduced to malate by malate dehydrogenase. The only enzyme in *D. acetivorans* that could possibly catalyze this ATP-independent cleavage is citrate synthase. In the cell extracts of *D. acetivorans*, fumarate reductase activity, which is another enzyme characteristic of the rTCA cycle, was also detected, and the regulation of carbon flow was proposed to occur as follows: the presence of H_2_ changes the flow toward the roTCA cycle or drives the exogenous acetate from the TCA cycle in the oxidative direction [12].

Zhang et al. [102] also identified the roTCA pathway in *Geobacter sulfurreducens*. Until then, it was believed that this group of bacteria was unable to fix carbon. However, the identification of the roTCA cycle in this bacterium suggested that it may grow chemolithoautotrophically. The authors used genomic analysis to investigate whether *G. sulfurreducens* could fix inorganic carbon, and serially transferred the strain to a chemolithoautotrophic culture medium containing formate as an electron donor and carbon source, as well as iron as an electron acceptor. Furthermore, enzymatic assays also corroborated the carbon fixation, showing that citrate synthase can achieve citrate cleavage, which is necessary for the function of the roTCA cycle. These findings showed that *G. sulfurreducens* can fix CO_2_ through the roTCA cycle after adaptation; this previously unexplored metabolic pathway can be used for biotechnology and may elucidate previously unclear ecological functions for *Geobacter* [102].

## Development of synthetic pathways for carbon fixation

The global energy crisis and greenhouse effect have impeded the sustainable development of society [4]. Thus, reducing the emission of CO_2_, which causes the greenhouse effect, is an urgent task. However, increasingly efficient carbon-fixation processes are also required because the rate of natural carbon fixation is inadequate to balance the CO_2_ released from industries. Carbon fixation performed by photosynthetic organisms allows the recycling of greenhouse gas emissions into high value-added products. However, the dependence on light to drive carbon fixation can be limiting for industrial chemical synthesis [103]. Although natural carbon-fixing processes are not feasible for use in industrial production, they offer a variety of carbon-fixing enzymes and corresponding pathways, opening options for artificial engineering [4]. Thus, in recent years, synthetic carbon-fixation pathways have been developed to increase the efficiency of CO_2_ fixation and reduce the loss of CO_2_ during this process.

## Crotonyl-(CoA)/ethylmalonyl-CoA/hydroxybutyryl-CoA (CETCH) cycle

The CBB cycle is the most prevalent CO_2_ assimilation mechanism in the biosphere and is found in all plants, algae, and some prokaryotes. However, its efficiency is limited for natural carbon fixation, rendering the productivity of this path extremely low despite its widespread ecological success owing to the availability of free energy through sunlight.

Schwander et al. [104] described an *in vitro* synthetic carbon-fixation pathway called the CETCH cycle, which surpasses natural CO_2_ fixation in its efficiency. This pathway involves 17 enzymes that convert CO_2_ into organic molecules at a rate of five nanomoles of CO_2_ per minute, using two CO_2_ molecules along with two ATPs and three NAD(P)H cofactors, which are the same energy carriers as for the oxygenic photoautotrophs.

The CETCH cycle was realized through a combinatorial approach involving enzyme engineering and metabolic review that was described as a radical and reductionist approach. This CO_2_-fixation cycle was assembled from its main components in an ascending manner. First, a comparison was made between the kinetic and biochemical properties of all the known classes of carboxylases, and based on these analyses, the enzymes CoA-dependent carboxylases and enoyl-CoA carboxylases/reductases (ECRs) were chosen. ECRs are found in Alphaproteobacteria and *Streptomyces*, whose carboxylation activities surpass that of the propionyl-CoA carboxylase involved in the 3-HP bicycle, HP-HB cycle, and RuBisCO carboxylation [105], [106]. In comparison with other carboxylases, ECRs work with a broad spectrum of substrates, are insensitive to oxygen, do not accept oxygen as a substrate, and catalyze CO_2_ fixation with a high catalytic efficiency [107]. The CETCH pathway converts two CO_2_ molecules from proprionyl-CoA to form a glyoxylate molecule through 13 main reactions catalyzed by 17 enzymes belonging to nine different organisms from four domains of life, including plants, humans, and microbes (Bacteria and Archaea), thereby indicating the potential for natural diversity.

The CETCH cycle shares four reactions and five intermediates with the HP-HB cycle [108]. At the time of writing this manuscript, the CETCH cycle was not implemented in a living host (neither a heterotrophic host, nor a photoautotrophic host). Indeed, balancing the expression of 17 separate enzymes in a heterologous system is challenging even for the most genetically tractable organisms. Thus, the development of artificial carbon-fixation pathways may be limited by the gap between theoretical predictions and their experimental realization in synthetic biology. Moreover, attempts to synthesize new metabolic pathways in living organisms are challenging because of the limited understanding of the interactions between enzymes in heterologous systems [104]. Although the CETCH cycle presents attractive features for implementation *in vivo*, its synthetic engineering in microorganisms remains undemonstrated [109].

*Synthetic reductive glycine pathway* (rGlyP).

Carbon fixation can be implemented through assimilation routes based on natural or synthetic formats [110]. In recent synthetic biology studies, a formate-assimilation pathway, called the rGlyP pathway, that supports carbon fixation if coupled with CO_2_ reduction has been demonstrated [93], [110], [111]. The rGlyP cycle is structurally similar to the most efficient carbon-fixation cycle known, i.e., the WL route. Both routes start with the reduction of CO_2_, followed by the conversion of formate into tetrahydrofolate, and progressing to further reduction. This cycle uses an enzymatic complex to assimilate carbon and generate CO_2_ compounds and convert them to pyruvate by condensation with another unit of carbon [110]. Formate is the product of CO_2_ reduction by an electron pair and is considered to be the simplest organic compound that can supply cells with carbon and reducing power [110]. An efficient carbon-fixation pathway is one that combines CO_2_ reduction with carboxylation, as shown by the rGlyP cycle [93]. Thus, the synthetic rGlyP pathway represents an efficient route because of the combination of ATP-free CO_2_ reduction, carboxylation, and versatility.

The rGlyP route seems be a promising pathway for the assimilation of formate and other sustainable C1-feedstocks and could be applicable in future biotechnology-based attempts [112]. Modular engineering was used to implement the rGlyP for supporting synthetic formatotrophic growth as well as for growth on methanol in *Escherichia coli* [99]. The rGlyP was divided into four metabolic modules: 1) the C1 module (C1M), which consisted of the enzymes formate-THF ligase, methenyl-THF cyclohydrolase, and methylene-THF dehydrogenase from *Methylobacterium extorquens* that together converted formate into methylene-THF; 2) the *C*2 module (C2M), which consisted of endogenous enzymes of the GCS (GcvT, GcvH and GcvP) that converted methylene-THF with CO_2_ and ammonia to form glycine; 3) the C3 module (C3M), which utilized serine hydroxymethyltransferase and serine deaminase that condenses glycine and methylene-THF to generate serine and pyruvate; and 4) the energy module, which used formate dehydrogenase from *Pseudomonas* sp. [99]. This approach was used to redesign the central carbon metabolism of the model in *E. coli* that supported the growth of the bacteria with one carbon using rGlyP, and the growth in methanol and CO_2_ was achieved by the additional expression of a methanol dehydrogenase. Biologically adapted microbial growth using formate and methanol has been investigated by the synthetic biology community in recent years [113]. Thus, this synthetic route appears to be finally becoming possible.

## Formaldehyde fixation 1 - the formolase pathway

Even though formaldehyde is considered an organic carbon, sections 4.3 and 4.4 were included to discuss the complementary synthesis mechanisms of carbon fixation. To shorten the process of using carbon and accelerate growth, an artificial synthetic route the formolase pathway—was computationally designed [17], and the computationally designed enzyme, called formolase, performs a reaction that catalyzes carboligation by directly fixing the units of one carbon in units of three carbons that feed the central metabolism. By integrating formolase with various naturally occurring enzymes, a new pathway for carbon fixation has been created that assimilates units of a carbon via formate. The enzyme formolase catalyzes the carboligation of three formaldehyde molecules with one carbon into one dihydroxyacetone molecule with three carbons [5].

## Formaldehyde fixation 2 - synthetic acetyl-CoA (SACA)

The efficiency of carbon fixation can also be improved during the synthesis of acetyl-CoA, which is one of the central precursors involved in the biosynthesis of various products [114]. Recently, a synthetic route for acetyl-CoA (SACA) was designed using a glycolaldehyde synthase and an acetyl phosphate synthase and was introduced in *E. coli* [6]. In the reported SACA pathway, two formaldehyde molecules were transferred to an acetyl-CoA molecule in just three steps by using two ATP and two NADH molecules. First, the formaldehyde was condensed into glycolaldehyde by an glycolaldehyde synthase. Subsequently, the glycolaldehyde was converted to acetyl phosphate by an acetyl phosphate synthase. The glycolaldehyde synthase and acetyl phosphate synthase were selected and designed to increase their catalytic efficiency with their new substrates. At the end of the cycle, the acetyl phosphate group was replaced by CoA in a process catalyzed by a phosphate acetyltransferase, and both *in vitro* and *in vivo* production of acetyl-CoA was achieved. The pathway exhibited high carbon-fixation capabilities [115], [116]. The SACA pathway works with five reaction steps and enables acetyl-CoA production from formaldehyde; however, employing this route in *E. coli* can be challenging due to the low enzyme activity of glycolaldehyde synthase engineered, but glycolaldehyde synthase seems to show improved affinity to substrate formaldehyde [117], [118]. Another question is whether the SACA pathway requires high intracellular concentration of formaldehyde, which can be toxic for the cells [118].

To overcome the deficiency of the CBB cycle for the efficient synthesis of acetyl-CoA, Yu and collaborators [119] designed the synthetic malyl-CoA-glycerate (MCG) pathway. The CBB cycle has not evolved for optimal production of acetyl-CoA; when 3-phosphoglycerate, which is the product of the CBB cycle, is converted to acetyl-CoA, one fixed carbon is lost as CO_2_ [119]. In the synthetic MCG, a C_3_ sugar is converted into two acetyl-CoA by fixing only one carbon or by assimilating glyoxylate without the loss of carbon in photorespiration. In this synthetic carbon-fixation pathway, two oxaloacetate molecules are reduced to malate, which is then converted to malyl-CoA and later splits into two acetyl-CoAs and two glyoxylates. These two acetyl-CoAs are the final products of the pathway, and the two glyoxylates are used to regenerate PEP. The MCG pathway converts a PEP and HCO_3_ into an acetyl-CoA using three ATPs and three NADHs. The functionality of the MCG pathway was first demonstrated *in vitro* and in *E. coli*. Later, the pathway was implemented in the photosynthetic strain *Synechococcus elongatus* PCC7942, which showed an increase in the intracellular pool of acetyl-CoA as well as an improvement in the assimilation of HCO_3_ [119]. Recently, a system of CO_2_ fixation oxygen insensitive, self-replenishing with opto-sensing was demonstrated through the junction of the MCG pathway and synthetic reductive glyoxylate and pyruvate synthesis *in vitro.* This system produced acetyl-CoA, pyruvate, and malate from CO_2_, and its self-replenishing feature allowed every intermediate of system to be produced from CO_2_ [120].

## Engineering RuBisCO for enhanced CO_2_ fixation

The low efficiency of carbon fixation in autotrophs, limited by the enzyme RuBisCO, is a biotechnological shortcoming that has led to numerous studies focused on genetic engineering with different strategies to optimize the carboxylase function of this enzyme. RuBisCO is considered the most abundant enzyme on Earth, consituting 50 % of the total soluble protein in the chloroplasts of a C_3_ plant or in bacteria that use this cycle [121], [122]. In addition, RuBisCO also catalyzes a competitive reaction with oxygen and initiates the photorespiration process, which leads to a loss of fixed carbon and involves significant consumption of ATP to fix CO_2_. However, in comparison with other enzymes in the CBB cycle, RuBisCO has a low turnover rate, implying that large amounts of this enzyme are needed to sustain the efficiency of CO_2_ assimilation [123].

Improving RuBisCO by using bioengineering is a complex challenge due to two key aspects: 1) identification of the structural changes that promote performance and 2) identification of the ways to efficiently transplant these changes into RuBisCO within a target organism. Additionally, this task requires a satisfactory understanding of the regulatory pathways of the chloroplast gene, as well as the complex nature of catalysis and biogenesis promoted by this enzyme [32], [124].

Despite the challenges in production, substantial progress has been made in bioengineering RuBisCO. In a previous study, the construction of a mutant RuBisCO was adopted as a strategy to improve the rate of carbon fixation in plants. Substitutions were made in the small subunit of the RuBisCO enzyme, thereby increasing the carboxylation activity by 85 % and enhancing the catalytic efficiency toward CO_2_ by 45 % [125]. According to that study, the RuBisCO mutant could still be transplanted into higher plants if they shared the same hexadecameric L8S8 structure.

Another strategy already used to improve RuBisCO performance is the replacement of native RuBisCO by an exogenous homolog. Lin et al. [126] worked on replacing the RuBisCO of tobacco plants with the functional RuBisCO of the cyanobacterium *Synechococcus elongatus* PCC7942 (Se7942). The native gene encoding the large RuBisCO subunit was knocked out, and the genes for the large and small subunits of Se7942 corresponding to a chaperone, RbcX, or an internal carboxysomal protein were inserted. The transformed plants were photosynthetically competent and supported autotrophic growth, and the respective forms of RuBisCO exhibited greater rates of CO_2_ fixation per unit of enzyme than the control plants [126].

The discovery of the new RuBisCO assembly factors (C_3_ and C_4_ plants) has provided next steps for improving this enzyme for higher plants [127], [128]. For example, improving CO_2_ assimilation now includes equipping C_3_ plants with a CO_2_ concentration mechanism and generating alternative metabolic pathways to bypass the oxygenation [129], [130].

Gene editing tools, such as chemical and physical mutagenic agents, that operate on nuclear DNA can be used to modify the family of multiple *rbc*S genes or insert new *rbc*S copies and are commonly applicable on various cultures [131]. Because the transformation of the chloroplast is viable in only a few species, modifying the *rbc*L gene in the chloroplast genome is excessively complicated [132]. Thus, the implementation of RuBisCO in practical applications has necessitated the establishment of chloroplast transformation in several varieties of plants [133].

## Mixotrophic-mediated inorganic carbon uptake enhancement

Many photosynthetic eukaryotic microorganisms are capable of consuming organic carbon sources in addition to CO_2_. Higher plants arose from the first photosynthetic flagellated protists, which acquired a cyanobacterial endosymbiont that led to the chloroplast in photosynthetic eukaryotes. As these organisms are found in every environment, they possess the ability to consume organic carbon in the absence of light and CO_2_, exhibiting an advantageous metabolic capacity. Within the green algae, the genus of *Chlorella* is ubiquitous around the world, and *C. vulgaris* has been generally regarded to show a safe status as an alternative protein-rich biomass, nutraceutical, and source of bio-compounds.

This organism can be fermented like yeasts on simple sugars as a carbon source [134], [135]. *Chlamydomonas* is another genus of green algae, known for only being able to metabolize organic acetic acid via the glyoxylate cycle [136], whereas in the red algae, the extremophile *Galdieria sulphuraria* is known for its ability to metabolize over 50 different carbon sources [137]. The consumption of glucose in algae has been observed to correlate with a reduction in photosynthetic activity, in which the heterotrophic growth is favored over the autotrophic growth [138]. However, glycerol addition to mixotrophic cultures of the diatom *Phaeodactylum tricornutum* or *G. sulpheraria* increases their overall growth rates, resulting in higher growth rates in the presence of light and CO_2_. *Chlamydomonas reinhardtii* demonstrates marked improvements in growth behavior in photo-mixotrophic conditions of high-CO_2_ cultures fed with acetic acid [139], [140]. Each of these organic carbon sources is metabolized through a different pathway and clearly stimulates different cellular responses. For example, although acetic acid mixotrophy reduces the chlorophyll content in exponential-phase *C. reinhardtii* cultures, the overall photosynthetic rates and biomass formation are improved [140]. We postulate that large amounts of organic carbon precursors can increase the amounts of CBB intermediates and, concomitantly, improve their ability to sequester CO_2_ [140].

Algal cultivation is generally aimed at maximizing biomass production for a range of different applications, and now, with the advent of synthetic biological techniques, the engineering possibilities in these hosts have increased [141], [142]. Examples of engineered algae as sources of polyamines, isoprenoids, and recombinant proteins have been demonstrated using mixotrophic cultivation strategies [143], [144], [145]. Algae offer a unique platform for waste carbon conversion, and the study of mixotrophic carbon uptake enhancement can increase the yield of CO_2_ and provide desired products via waste-stream conversions.

## Carbon fixation in thermophilic microorganisms

Thermophiles are microorganisms that can survive above the typical thermal limits of life, in inhospitable environments [146]. Although the temperature range for these microbes varies between 45 °C and 80 °C, the hyperthermophiles can survive even at temperatures above 80 °C [147], [148]. They are found in volcanic areas, hydrothermal vents, solfatara fields, soils heated by steam, geothermal plants, and deserts [20]. Archaea, Bacteria, and Eukarya are the three domains of life in which extremophiles are found [149]. These microorganisms have genetic and physiological complexes that assist them in tolerating the environmental changes and adapting and repairing the damage caused by extreme environmental disturbances [150], [151].

Thermophiles have attracted interest for biotechnological applications requiring whole cells, pure cultures, or consortia, and for applications wherein their macromolecules, metabolites and extreme enzymes are used [152], [153]. Proteins and cell membranes with thermostable capabilities are not denatured at high temperatures; some can also resist proteolysis, making them highly desirable for industrial applications [20], [154]. The discovery of the polymerase enzyme in the thermophiles *Thermus aquaticus* and *Pyrococcus furiosus* significantly influenced and revolutionized the use of the polymerase chain reaction owing to its stability and reasonable cost [62], [155], [156].

Life is believed to have originated in a hydrothermal environment, and chemoevolution began in volcanoes through a transition metal-catalyzed autocatalytic carbon-fixation cycle [77]. Three of the six carbon-fixation pathways were found in thermophilic microorganisms, in the Archaea and Bacteria domains. For example, approximately 50 % of the bacterial cellular carbon originates from pyruvate/PEP (a gluconeogenic substrate that forms cell wall components and nucleotides), followed by acetyl-CoA (approximately 30 %), oxaloacetate (approximately 13 %), and alpha-ketoglutarate (approximately 7 %) [157]. The 3-HP cycle was found in the bacterium *Chloroflexus aurantiacus*, known as a thermophilic filamentous anoxygenic photoheterotrophic bacterium, that was initially isolated in hot springs and found to develop at temperatures between 45 °C and 75 °C [158], [159].

The carbon-fixation pathways HP/HB and DC/HB have been found in thermophilic Archaea. The HP/HB cycle was found in the Archaea *Metallosphaera sedula*, which grows autotrophically at 73 °C [160], and the DC/HB cycle was reported for the hyperthermophilic Archaea *Ignicoccus hospitalis* [79], [161]. *Ignicoccus* was isolated from the submarine hydrothermal ventilation systems, which are anaerobic, hyperthermophilic, always chemolithoautotrophic, and exhibit an optimal growth temperature of 90 °C [162].

In contrast, the CBB cycle occurs in several thermophiles, but never in hyperthermophiles, showing a temperature limit between approximately 70 °C and 75 °C [8]. This temperature limit of the cycle can be explained by the heat-induced instabilities of some intermediates in the cycle. Such instabilities are mainly observed in glyceraldehyde-3-phosphate, which produces toxic methylglyoxal at high temperatures [163]. The thermophilic bacteria *Thermosulfidibacter takaii* ABI70S6T showed a greatly efficient and reversible citrate synthase that needs reduced ferredoxin in the pathway [12], [164]. Until this discovery, it was believed that the CBB cycle can be reversed only to cleave citrate and fix CO_2_ autotrophically, however, this can be achieved only with alternative enzymes (e.g., citrate lyase). According to the authors, this question cannot be answered by metagenomics approach, but classical biochemistry can fill the gaps between genome sequences and organism phenotypes.

Although most species that use the rTCA cycle are mesophilic, the representatives of Aquificae are thermophiles that grow best at ≥ 70 °C, whereas *Aquifex aeolicus* grows best at temperatures up to 95 °C [23]. *Hydrogenobacter thermophilus,* a member of the phylum Aquificae, invests additional ATP in the conversion of 2-oxoglutarate to form isocitrate by the combination of an irreversible biotin-dependent 2-oxoglutarate carboxylase and a noncarboxylating isocitrate dehydrogenase [165]. This process possibly becomes effective and irreversible at high temperatures [23]. A recent fixation cycle describing the roTCA cycle was discovered in a thermophilic Deltaproteobacterium, *Desulfurella acetivorans* (a sulfur-reducing thermophile showing optimum growth at 52 °C-57 °C), through metagenomic analysis. In addition to the carbon-fixation pathways, the key enzymes involved in these routes have also been described in these thermophiles. Aoshima et al. [166] reported a new key enzyme, called the citryl-CoA synthetase, which was isolated from *Hydrogenobacter thermophilus*. This enzyme, which is involved in the rTCA cycle, could reportedly catalyze the first citrate cleavage step.

Carboxydotrophic bacteria are also found in geothermal environments, suggesting that CO-oxidizing Bacteria and Archaea perform an important role in thermophilic environments [167]. For example, acetogens and methanogens can survive in thermophilic systems and use CO as a carbon source [168]. In the WL pathway, CO is a key intermediate in the fixation of CO_2_ into acetyl-CoA in acetogens, methanogens, and sulfate-reducing bacteria [169], [170].

Recently, a novel Archaea phylum from terrestrial hot spring and deep-sea hydrothermal vent sediments that presented unique and versatile carbon cycling pathways was described [171]. Metagenome-assembled genomes were reconstructed from 15 Archaea genomes, and a new phylum classified as Brockarcheota was proposed. The Brockarchaeota are capable of mediating nonmethanogenic anaerobic methylotrophs via the tetrahydrofolate methyl branch of the WL pathway and the reductive glycine pathway. The members of this unique phylum exhibit a type of anaerobic methylotrophic metabolism that has not been described previously in Archaea. The methyltransferase process is composed of two key steps: first, specific methyltransferases break the C-R bonds in several substrates (e.g., methanol and trimethylamine) and transfer the methyl moieties to the subunit MtaC; then, the methyltransferase (MtaA for methanol and MtbA for methylamines) transfers the methyl group from the corrinoid protein to coenzyme M in the methanogens or to tetrahydrofolate in the acetogens [171], [172].

Brockarchaeota lack the key enzymes from the methyl and carbonyl branches of the WL pathway, which are involved in the transfer and reduction of the C_1_ fraction for methane production. Furthermore, Brockarchaeota do not encode the pyrroloquinoline quinone-linked methanol dehydrogenase pathway for aerobic methylotrophic reactions. This lack of methylotrophic pathways suggests that the new phylum metabolizes methanol and trimethylamine through the convergent action of the tetrahydrofolate (H_4_F) methyl branches of the WL and glycine-reduction pathways [171].

The phylum Brockarchaeota also presents an unknown pathway for butanol metabolism, since key enzymes involved in the fermentation of pyruvate to butanol are absent in their genomes [171]. Nevertheless, most of the 15 genomes encode a putative aldehyde dehydrogenase that can convert butyraldehyde to butyric acid. In addition, a putative enoyl-CoA hydratase/isomerase protein has been found to be involved in the further conversion of butyric acid to acetyl-CoA, which suggests an alternative pathway for the oxidation of butanol. Members of this phylum can also perform the following activities: (i) degrade complex carbon compounds such as xylan, (ii) assimilate formaldehyde and formate, (iii) use arsenate as an electron acceptor, (iv) possibly reduce sulfur during fermentative growth and produce H_2_S, and (v) possibly use group 3b [NiFe]-hydrogenases for the oxidation of H_2_ with NADP or NAD(P) as the electron acceptors [171], [172], [173].

In the early 1990 s, Gadkari et al. [174] described a new species of *Streptomyces,* which was a thermophilic-carboxidotrophic-chemolithoautotrophic microbe isolated from soil covering a burning charcoal pile, and named it *Streptomyces thermoautrotophicus* UBT1. In contrast to the other carboxidotropics, this strain is unique in its exclusive use of a lithotrophic substrate with a new CO dehydrogenase. Several decades later, MacKellar et al. [175] described a new strain of *Streptomyces thermoautrotophicus* (strain H1), which was isolated from another burning charcoal pile; furthermore, multiple CO dehydrogenase gene clusters were identified in the genomes of the strains UBT1 and H1 [176].

Recently, Volpiano et al. [176] proposed a new reclassification of the species and genus *Streptomyces thermoautrotophicus,* which was modified to *Carbonactinospora thermoautotrophica* based on its phylogenetic placement and distinctive phenotypes among *Actinomycetes*. Multiple genes related to carbon metabolism were found in this species, including ribulose bisphosphate carboxylase (*rbc*L), phosphoenolpyruvate carboxykinase, glucose/mannose-6-phosphate isomerase and PFK 6-phosphofructokinase. Thus, more investigations are necessary to identify the carbon path used by these thermophilic strains [176].

These findings indicate that thermophilic microorganisms are important for identifying new ways of fixing carbon. Thus, it is crucial to identify microorganisms that can participate in the global carbon cycle and provide alternatives for applications in various sectors of biotechnology.

## Challenges and perspectives

The main challenge underlying the manipulation and modification of carbon-fixation routes is the insufficient understanding of how the pathways perform together within the studied organism. The use of fossil fuel resources is one of the main causes of global warming, being strictly related to the increase of atmospheric CO_2_ concentration [110]. Natural photosynthesis can convert approximately 100 billion tons of CO_2_ into biomass annually; however, RuBisCO, which is the most abundant enzyme on Earth, itself has a limited carboxylation rate, indicating an extremely low productivity of this pathway [126]. Synthetic biologists have made numerous efforts to overcome these challenges and improve the specificity of RuBisCO. Natural carbon-fixation processes are often slow, increasing the difficulty in designing genetically modified autotrophic plants and organisms and improving native CO_2_ fixation [4], [110].

Since the discovery of other natural pathways of carbon fixation besides the well-known CBB cycle, studies have attempted to identify the existence of other pathways in organisms that were not previously thought to fix carbon. This has led to a better understanding of the functioning of the pathways and the catalytic power of key enzymes related to carbon fixation. In addition, these findings have raised the question of increasing the efficiency of already known pathways as well as redesigning new pathways of carbon fixation to naturally reduce the amount of anthropogenically emitted CO_2_.

The global objective is to achieve liquid zero carbon emissions by the year 2050 [177]. This emitted carbon can be fixed, stored, and converted into sustainable feedstock. The present review aimed to elaborate the natural carbon pathways, as well as highlight the synthetic pathways that have been developed over the last decades. Given the need for shedding light on these two subjects, we collected and centralized the available data on the recent research in this field. In recent years, different pathways have emerged to improve carbon-fixation efficiency, demonstrating the importance of this discussion. Furthermore, the most important step will be to apply these efficient pathways in hosts and make them functional.

Based on the recent progress made in this field, certain problems related to biological carbon fixation have been identified [4]. Due to the low activities of the key carbon-fixing enzymes, there are limitations on natural carbon-fixation pathways. However, the rapid advancements in microbiome studies and their related “omics” analyses have generated large amounts of data, from which novel and more efficient carbon-fixing enzymes can be identified. Natural carbon-fixation pathways have several shortcomings, with many reaction steps and low efficiencies. However, the development of synthetic biology permits the exploration of new natural carbon-fixation pathways and the design of novel synthetic carbon-fixation techniques. Another barrier is the low solar energy-absorption efficiency of carbon fixation, which significantly reduces the economic benefits. These limitations can be addressed by converting light to other sources such as electric energy and hydrogen for direct carbon fixation. Chemoautotrophs provide an alternative platform because they can be driven chemically by renewable H_2_ instead of light [103]. Electrical energy can also be applied to produce energy carriers like formate, hydrogen, carbon monoxide, methanol, and methane [178]. Li et al. [179] described *Ralstonia eutropha* H16, an autotrophic genetically engineered microorganism, that produced high levels of alcohol in an electric bioreactor using CO_2_ as the sole carbon source and electricity as the sole energy input. Another approach could be to improve light-utilization efficiency. Recently, Nürnberg et al. [180] discovered specific photosystems I and II in *Chroococcidiopsis thermalis* that can use energy from far-infrared radiation.

Due to the low efficiencies and limited scope for performing genetic modifications of the natural carbon-fixation systems, various researchers are exploring these systems using different ways; for example, introducing partial or total carbon-fixation pathways in heterotrophic chassis cells. Generally, heterotrophic microorganisms exhibit superior growth and production yields than autotrophic microorganisms [17]. The significant improvements in synthetic biology permit the engineering of novel functions and metabolic networks in heterotrophic microorganisms for innumerous biotechnological applications [178], [179], [180], [181], [182]. Novel genes mediating the CO_2_ fixation pathway in autotrophic microorganisms may apply negative influences on the regulatory networks in cells, such as carbon/nitrogen metabolism; however, they have been widely used as chassis to express natural CO_2_-fixation pathways in the model heterotrophic microbes *Escherichia coli* and *Saccharomyces cerevisiae* [183], [184].

In the near future, novel enzymes and entire pathways of carbon fixation are expected to be identified with rapid development of “omics” tools and microbiome projects [4]. These advancements will facilitate the development of feasible methods for changing and/or increasing the production of the desired product. Moreover, the use and availability of “omics” tools has progressed rapidly, highlighting the limitations of single techniques in dealing with the complexities of biological systems [185].

One of the future possibilities is the integration of data obtained using the “omics” tools with studies on metabolism and kinetics, classical biochemistry, and molecular biological techniques. In addition, the development of mathematical models to analyze biological processes on a micro-scale will allow us to predict their effects on a macro scale. In the near future, these suggested methods may facilitate the exploration, modeling, manipulation, and engineering of metabolic pathways in natural and synthetic microbial systems. According to Bar-Even et al. [115] and Fuchs [7], the number of known autotrophic pathways is expected to increase with the aid of genome searching and the application of synthetic biological tools. Notably, “omics” analyses have revolutionized the research on carbon fixation and unveiled the genomic black box. Nevertheless, for better results, these analyses must be linked to bench tests and classical biochemical analyses to obtain detailed and robust results because some resources may not be identified solely with bioinformatics tools.

Although carbon-fixation pathways have not yet been used at the industry level owing to the low catalytic efficiencies of the enzymes and the need for a significant energy input, studies of these routes are anticipated to provide a plethora of possibilities for biotechnological advances. The major challenge, i.e., the joining of carbon-fixation pathways with synthetic pathways due to the differences between the metabolism of autotrophs and heterotrophs that are used as chassis, can be resolved. Carbon-fixation pathways based on synthetic biology have already been described, and the energy supply and more efficient enzymes can be redesigned in these pathways [186].

## Conclusion (Remarks)

Carbon is often negatively associated with the anthropogenic emissions of greenhouse gasses; however, it is also an element of paramount importance for life and ecological processes. The knowledge of carbon-fixation cycles, in addition to ecological and evolutionary studies on the emergence of the first beings that inhabited Earth and metabolized carbon, can provide a detailed understanding of the enzymes and important intermediaries involved in these cycles.

The central carbon-metabolizing enzymes can be used for a variety of purposes; for example, artificial CO_2_ fixation pathways can be designed and implemented in suitable host organisms. Additionally, new solutions are possible for manipulating carbon-fixation pathways and increasing their final yields. The multidisciplinary integration of “omics”, synthetic biology, molecular biology, and biochemistry can enable the scientific community to describe new carbon-fixation pathways and predict important routes and enzymes involved in these processes. This integration may provide further guidance for overcoming the limitations of natural carbon-fixation pathways and allow the development of biotechnological applications.

Nevertheless, investigation of thermophilic microorganisms is crucial for understanding many evolutionary events. Overall, these microorganisms have been extensively investigated for developing biotechnological applications because of their metabolic capabilities; however, thermophiles remain underexplored with regard to the identification of novel natural routes of carbon fixation and the development of efficient synthetic pathways.

**Funding**

This study was funded by KAUST (BAS/1/1096-01-01). SSC was awarded scholarships from the Foundation for Research Support of the State of Rio de Janeiro (FAPERJ) and Coordination for the Improvement of Higher Education Personnel (CAPES).

**Compliance with Ethics Requirements**

*This article does not contain any studies with human or animal subjects*.

## CRediT authorship contribution statement

**Sulamita Santos Correa:** Conceptualization, Methodology, Writing – original draft, Writing – review & editing. **Junia Schultz:** Data curation, Writing – original draft, Writing – review & editing. **Kyle J. Lauersen:** Validation, Writing – review & editing. **Alexandre Soares Rosado:** Supervision, Writing – review & editing.

## Declaration of Competing Interest

The authors declare that they have no known competing financial interests or personal relationships that could have appeared to influence the work reported in this paper.
